# Supplementary material for: Early Prediction of Dementia Using Feature Extraction Battery (FEB) and Optimized Support Vector Machine (SVM) for Classification
Source: Biomedicines. 2023 Feb 2;11(2):439. doi: 10.3390/biomedicines11020439 (PMC9953011; doi:10.3390/biomedicines11020439)
Supplement: Supplementary file 1 [file biomedicines-11-00439-s001.zip › biomedicines-2192800-supplementary.pdf]

Supplementary Material S1 - Description of all input variables

Table S1: List of the input variables with description, possible values and number of missing values

Variable Type	#	Variable	Description	Values	# Missing Values
Basic	1	Gender	Subject's gender	• Male<br>• Female	0
	2	Age	Subject's age	Numeric	0
	3	BMI	Subject's BMI	Numeric	0
Social	4	Education	Subject's Highest level of education	• Unfinished elementary school<br>• Elementary school<br>• High school<br>• Vocational training<br>• University<br>• Doctorate	51 (5.2%)
	5	Religion	Do you have a religious belief?	• Yes<br>• No	57 (5.8%)
	6	Religious Activities	"Do you participate in religious activities?	• Not at all<br>• Sometimes<br>• Often	39 (4%)
	7	Voluntary Association	"If you are a member of a voluntary association, could you say that you feel strong associations with this association and its members?"	• Not a member of the association<br>• Highly<br>• To some extent<br>• Not especially<br>• Not at all	56 (5.7%)
	8	Social Network	Assesses the subject's social network in terms of personal relationships and social interactions. See "Description 1"	• Very bad social network<br>• Bad social network<br>• Normal social network	57 (5.8%)
	9	Support Network	Assesses the subject's support network in terms of people that can help them with life issues. See "Description 2"	• Numeric	53 (5.4%)
	10	Loneliness	Asses the subject's feeling of loneliness. See "Description 3"	• Very high level of loneliness<br>• Some level of loneliness<br>• Normal level of loneliness	76 (7.8%)
Life Style	11	Exercise	Frequency of light exercise in last 12 months	• Wheelchair or balance problems<br>• Never<br>• Once a month<br>• 2-3 times a month	99 (10.1%)

				• More than 3 times a month<br>• Every day	
	12	Alcohol_Consumotion	How often do you drink alcohol	• Never<br>• Once a month or more rarely<br>• 2-4 times a month<br>• 2-3 times a week<br>• 4 or more times a week	46 (4.7%)
	13	Alcohol_Quantity	How many glasses do you drink on a typical day when you drink alcohol?	• Do not drink alcohol<br>• 1-2<br>• 3-4<br>• 5-6<br>• 7-9	94 (9.6%)
	14	Working 65	When did you stop working?	• Stopped working before 65 years	0
	15	Present Smoker	Do you smoke?	Numeric	34 (3.5%)
	16	PastSmoker_cigarsDay	If you have quitted smoking. How many cigarettes/day did you smoke on average before you stopped?	Numeric	63 (6.4%)
	17	Social Activities	Asses the subject's engagement in sociocultural demanding activities in the past 12 months. See Description 4	Numeric	85 (8.6%)
	18	Physically Demanding Activities	Asses the subject's engagement in physically demanding activities in the past 12 months. See Description 5	Numeric	85 (8.6%)
	19	Leisure Activities	Asses the subject's engagement in leisure and hobby activities in the past 12 months. See Description 6	Numeric	96 (9.8%)
Medical History	20	Medications	Number of medications taken regularly by the subjects	Numeric	0
	21	Family History	Subject's family (first degree relatives) medical history of importance, regarding cardiovascular disease,	• Yes<br>• No	2 (0%)

			Parkinson’s disease and dementia		
	22	Infract	Subject’s history of infarct	• Yes<br>• No	8(0%)
	23	Arrhythmia	Subject’s history of arrhythmia	• Yes<br>• No	13 (1.3%)
	24	Heart failure	Subject’s history of heart failure	• Yes<br>• No	1 (0%)
	25	Stroke	Subject’s history stroke	• Yes<br>• No	6 (0%)
	26	TIARIND	Subject’s history of Transient Ischemic Attacks or Reversible Ischemic Neurological Deficit (TIA/RIND)	• Yes<br>• No	11 (1.1%)
	27	Diabetes_type1	Subject’s history of Diabetes_type1	• Yes<br>• No	1(0%)
	28	Diabetes_type2	Subject’s history of Diabetes_type2	• Yes<br>• No	2 (0%)
	29	Thyroid Disease	Subject’s history of thyroid disease	• Yes<br>• No	5 (0%)
	30	Cancer	Subject’s history of cancer	• Yes<br>• No	1 (0%)
	31	Epilepsy	Subject’s history of epilepsy	• Yes<br>• No	0
	32	Atrial Fibrillation	Subject’s history of atrial fibrillation	• Yes<br>• No	75 (7.7%)
	33	Ischemic Signs	Subject’s history of ischemic signs	• Yes<br>• No	76 (7.8%)
	34	Parkinson’s	Subject’s history of Parkinson’s	• Yes<br>• No	3(0%)
	35	Depression	Subject’s history of depression	• Yes<br>• No	0
	36	Other Psychiatric Disease	Subject’s history of psychiatric disease	• Yes<br>• No	5(0%)
	37	Snoring	Subject’s history of snoring	• Yes<br>• No	3(0%)
	38	Sleep Apnea	Subject’s history of sleep apnea	• Yes<br>• No	7(0%)
	39	Hip Fracture	Subject’s history of hip fracture	• Yes<br>• No	10 (1.0%)
	40	Head Trauma	Subject’s history of head trauma	• Yes<br>• No	8(0%)
	41	Developmental Disabilities	Subject’s history developmental disabilities	• Yes<br>• No	0
	42	High Blood Pressure	Subject’s history of blood pressure	• Yes<br>• No	11 (1.1%)
Blood Test	43	HB	Blood test analysis of the amount of hemoglobin in the blood (g/L)	Numeric	19 (1.9%)
	44	CRP	Blood test analysis of the amount of C-	Numeric	30 (3.0%)

			reactive protein in the blood (mg/L)		
Physical Examination	45	Pain	Have you had pain the last 4 weeks?	• Yes<br>• No	53 (5.4%)
	46	Heart Rate_sitting	Subject's heart rate in beats per minute, while sitting	Numeric	16 (1.6%)
	47	Heart Rate_Lying	Subject's heart rate in beats per minute, while lying	Numeric	25 (2.5%)
	48	Blood Pressure_Right	Subject's systolic blood pressure measured on the right arm, while lying (mmHg)	Numeric	21 (2.1%)
	49	Hand Strength_Right	Subject's righthand strength in Newtons, during an interval of 10s, measured by the Grippit instrument.	Numeric	85 (8.6%)
	50	Hand Strength_Left	Subject's left hand strength in Newtons, during an interval of 10s, measured by the Grippit instrument.	Numeric	88 (8.9%)
	51	Rise_Safe	Does it feel safe for you to rise from a chair without using your arms?	• Yes<br>• No, it feels unsafe<br>• Cannot stand up	63 (6.4%)
	52	Rise_How	Rising from the chair. How?	• Got up without using their arms<br>• Got up, but using their arms<br>• Tired but couldn't<br>• Not tried for security reasons<br>• Not tried as there was no suitable chair<br>• On a wheelchair	67 (6.8%)
	53	WeightLoss_3months	Any weight loss during the last 3 months?	• Yes, more than 3 kg<br>• Don't know<br>• Yes, more than 1 kg, but less than 3 kg<br>• No weight loss	16 (1.6%)
	54	Standing Test_Right	Single led standing with right leg. Best value in seconds of three tries.	Numeric	72 (7.3%)
	55	Standing Test_Left	Single led standing with left leg. Best value in seconds of three tries.	Numeric	72 (7.3%)
	56	Dental_Prothesis	Assessment via x-ray of the subject's jaws in	• Only own teeth	4(0%)

			regards to their own teeth and prosthesis	• Own teeth and removable dentures<br>• Own teeth as well as removable prosthesis in one tooth jaw, or toothless and whole prosthesis in one tooth jaw<br>• Completely toothless<br>• Completely toothless and complete denture in one or both jaws with implants	
	57	Dental_TeethNumber	Assessment via X-ray of the subject's jaws in regards to the number of own teeth.	Numeric	96 (9.8%)
Psychological	58	Memory loss	Assessment of the subject's memory in daily life situations. See Description 7	Numeric	91 (9.3%)
	59	Memory Decline	Do you think your has gotten worse?	• No<br>• Somewhat<br>• A lot	10 (1.0%)
	60	Memory Decline 2	Does anyone in your circle think that your memory has gotten worse	• Yes<br>• No	18 (1.8%)
	61	Abstract Thinking	Explain the following phrase: The apple does not fall from the tree	• Wrong answer<br>• Wrong, only concrete answer<br>• Wrong abstract answer<br>• Right answer	46 (4.7%)
	62	Personality Change	Assess if the subject experienced changes regarding personality traits. Description 8	• Yes<br>• No	19 (1.9%)
	63	Identity	First the subject is asked questions about their identity: first name, Last name, year of birth, date of birth and age. Then S_Psychological_Identity index is calucuated as the sum of correct answers given by the subject.	Numeric	6(0%)

Health Instruments	64	SOC	Sense of Coherence [40]: assesses the subject's comprehensibility (how they perceive events as making logical sense), manageability (how they feel they can cope with situations), and meaningfulness (how they feel that life makes sense and challenges are worthy overcoming)	Numeric	56 (5.7%)
	65	DigitSpan_Forward	Forward Digit Span Test[41]: The tester say to the subject a sequence of numbers and the subject has to repeat it in the way they hear it. It starts up to two 3-number sequence going up to two 9-number sequence. Each correct sequence said by the subject counts as 1 point.	Numeric	12 (1.2%)
	66	DigitSpan_Backwards	Forward Digit Span Test [41]: The tester say to the subject a sequence of numbers and the subject has to repeat it backwards. It starts up to two 3-number sequence going up to two 9-number sequence. Each correct sequence said by the subject counts as 1 point.	Numeric	14 (1.4%)
	67	Livingston	Livingston Index [42]: a sleep disorder scale, composed of eight items regarding difficulty falling asleep or staying asleep, sleep medication usage, sleep interrupted at night, moods or tension, difficulty sleeping owing to pain or itching, inability to return to sleep after walking at night, waling up tpp early, or feeling tired more than two hours a day.	• No sleeping problem<br>• Presence of sleeping problem	31 (3.2%)

	68	EQ5D	EuroQol (EQ-5D) Index [43]: a generic instrument (non-disease specific) that aims to assess physical, mental and social functioning. The instrument is filled by the subjects who describes their own health-related quality of life in regards to mobility, self-care, usual activities, pain/discomfort and anxiety/distress. We used a dichotomized version of EQ-5D by the lower quartile index values.	• High quality of life<br>• Low quality of life	77 (7.9%)
	69	Index_Katz	Katz Index of Independence in Activities of Daily Living (ADL) [44]: Assesses functional status of the subject in regards to their capacity to perform activities of daily living independently.	• Severe impairment<br>• Moderate impairment<br>• Full function	17 (1.7%)
	70	IADL	Lawton Instrumental Activities of Daily Living (IADL) [45]: Assesses independent living skills of the subjects. Considers more complex skills than the ADL index.	• Dependent<br>• Independent	21 (2.1%)
	71	MMSE	Mini-mental State Examination (MMSE) [46]: Assess the cognitive aspects of mental functions.	Numeric	5(0%)
	72	ClockTest_Sum	Clock Drawing test [47]: Assesses cognitive impairment of a subject, in regards to verbal understanding, memory, spatially coded knowledge and construction skills. The 10-point score version was used in this study.	Numeric	37 (3.7%)
	73	MCS 12	Dichotomized Mental Composite Score of the SF-12 Health Survey [48]. The SF-12 is	• Low level of health<br>• High level of health	98 (10.0%)

			composed of 12 weighted questions that assess mental and physical functioning, and health-related quality of life. The Mental Composite Score is calculated from the designated questions and compared to the age-specific mean. An age-specific mean difference score of -5.5 points indicates a low level of health.		
	74	PCS 12	Dichotomized Physical Composite Score of the SF-12 Health Survey [48]. The SF-12 is composed of 12 weighted questions that assess mental and physical functioning, and health-related quality of life. The Physical Composite Score is calculated from the designated questions and compared to the age-specific mean. An age-specific mean difference score of -5.5 points indicates a low level of health.	• Low level of health<br>• High level of health	98 (10.0%)
	75	CPRS	Comprehensive Psychopathological Rating Scale [49]: it assesses the psychiatric state of the subject as to their level of depression.	• Absence of depression<br>• Mild depression<br>• Moderate depression<br>• Severe depression	13 (1.3%)

Description 1: Social Network
This is a categorical index that assesses the subjects' social network into one of the following categories: "Very bad social network", "Bad social network" and "Normal social network". It is built upon the questions described below, whose alternatives are attributed to a value held in parenthesis. The sum score of the values determines the category, as the following: a sum score of 5 characterizes a "Very bad social network"; a sum score in the range of 1 to 4 characterizes a "Bad social network"; a sum score of 0 characterizes a "Normal social network".

Questions:

- Do you think your number of friends is enough?
  - Too few (1)
  - Enough (0)
  - Too many (0)

- How many people do you think you know well and can talk about most of the time?
  - No one (1)
  - 1-3 (1)
  - 4-6 (0)
  - 7-9 (0)
  - 10-15 (0)
  - 16-30 (0)
  - More than 30 (0)

- Do you have someone who you feel you can be yourself in, who accepts you with all your merits and flaws?
  - Yes, without a doubt (0)
  - Yes, probably (0)
  - No, probably not (1)
  - Not at all (1)

- Do you feel close to your family (other than your husband, spouse, partner and children)?
  - Missing relatives (1)
  - Highly (0)
  - To some extent (0)
  - Not especially (1)
  - Not at all (1)

Description 2: Support Network
This index was built as the sum score of the questions' value regarding the subjects' social support network. The highest the score the worse social support network the subject has. The alternatives for the questions (with their respective values in parenthesis) are the following: Yes, without a doubt (1); Yes, probably (2); No, probably not (3); Not at all (4).

Questions:

- Can you get help from someone or someone in case of illness or other practical problems?
- Do you know someone or someone who can help you to write an official letter or appeal a government decision?
- Do you know that you have someone or someone who can provide you with proper personal support to cope with the stress and the problems of life?

Description 3 — Loneliness
This is a categorical index that assesses the subjects' felling of loneliness into one of the following categories: "Very high level of loneliness", "Some level of loneliness" and "Normal level of loneliness". It is built upon the questions described below, whose alternatives are attributed to a value held in parenthesis. The sum score of the values determines the category, as the following: a sum score of 4 characterizes a "Very bad social network"; a sum score in the range of 1 to 3 characterizes a "Some level of loneliness"; a sum score of 0 characterizes a "Normal level of loneliness".

Questions:

- Do you feel lonely?
  - Yes, often (1)
  - Yes, sometimes (0)
  - No, rarely (0)
  - No, never (0)

- When you look back on the last five years of your life, which of the following options best suits you?
  - I have not felt loneliness at any time in the past 5 years (0)
  - I have experienced occasional occasions with loneliness (0)
  - I have experienced recurrent periods of loneliness (1)
  - I have lived with a more or less constant feeling of loneliness (1)

- Do you feel a strong affinity with your local community?
  - Highly (0)
  - To some extent (0)
  - Not especially (1)
  - Not at all (1)

- Are you in a group of friends who have or do something in common?
  - Yes (0)

- No (1)

Description 4: Social Activities
The subjects were asked if they engaged in the following sociocultural activities in the past 12 months from the date, they responded the questionnaire: “Cinema, theatre, or concert”; “Restaurant, cafe, or pub”; “Church or religious meetings”; and “Study circle or course of some kind”. The possible answers were “yes” or “no”. The “Social Activities” index was built as the number of “yes” answers given by the subject.

Description 5: Physically Demanding Activities
The subjects were asked if they engaged in the following physically demanding activities in the past 12 months from the date, they responded the questionnaire: “Gardening”; “Taking walks outside”; “Picking berries or mushrooms”; “Hunting or fishing”; “Knit, weave or sew”; “Painting, drawing or sculpting"; “Home repairs"; and "Repairing cars or other mechanical equipment”. The possible answers were “yes” or “no”. The “Physically Demanding Activities” index was built as the number of “yes” answers given by the subject.

Description 6: Leisure Activities
The subjects were asked if they engaged in the following leisure activities in the past 12 months from the date, they responded the questionnaire: “Reading the newspaper”; “Reading magazines”; “Reading books”; “Watching television”; “Playing games or cards”; “Playing musical instruments"; “Listening to music"; and “Using the internet or playing computer games”. The possible answers were “yes” or “no”. The “Leisure Activities” index was built as the number of “yes” answers given by the subject.

Description 7: Memory Loss
This index was built as the sum score of the questions' value about the subjects' memory in daily life situations. The highest the score means the highest memory decline of the subject. The alternatives for the questions (with their respective values in parenthesis) are the following: Never (1); Rarely (2); Sometimes (3); Often (4); Always (5).

Questions:
- Do you happen to come to the store and have forgotten what to trade?
- Do you have trouble remembering what happened the day before?
- Do you lose or place things?
- Do you find it hard to know where you are?
- Do you find it difficult to find the right home / at department?
- Do you find it difficult to find the store / post office?
- Do you find it difficult to find in a foreign environment?

Description 8: Personality Change
The tester asks the subject if they felt like they changed in regards to the items in the questions below. A positive answer to 2 or more items defines a change in personality.

Questions:
- More or less talkative?
- More or less grumpy?
- More or less agitated?
- More or less withdrawn?
- More or less apathetic?
- More or less worried?
- More difficult than before to make decisions?
- More difficult than before to take the initiative?

References

40. Antonovsky, A. The structure and properties of the sense of coherence scale. *Soc. Sci. Med.* **1993**, *36*, 725–733, https://doi.org/10.1016/0277-9536(93)90033-z.
41. Wechsler, D. *WAIS-III Administration and Scoring Manual*; The Psychological Corporation: San Antonio, TX, USA, 1997.
42. Livingston, G.; Blizard, B.; Mann, A. Does sleep disturbance predict depression in elderly people? A study in inner London. *Br. J. Gen. Pr.* **1993**, *43*, 445–8.
43. Brooks, R.; Group, E. EuroQol: the current state of play. *Health Policy*, **1996**, *37*, 53–72, https://doi.org/10.1016/0168-8510(96)00822-6.
44. Katz, S. Assessing Self-maintenance: Activities of Daily Living, Mobility, and Instrumental Activities of Daily Living. *J. Am. Geriatr. Soc.* **1983**, *31*, 721–727, https://doi.org/10.1111/j.1532-5415.1983.tb03391.x.
45. Lawton, M.P.; Brody, E.M. Assessment of Older People: Self-Maintaining and Instrumental Activities of Daily Living. *Gerontologist* **1969**, *9*, 179–186, doi:10.1093/geront/9.3_Part_1.179.
46. Folstein, M.F.; Folstein, S.E.; McHugh, P.R. “Mini-Mental State”. A Practical Method for Grading the Cognitive State of Patients for the Clinician. *J. Psychiatr. Res.* **1975**, *12*, 189–198, doi:10.1016/0022-3956(75)90026-6.
47. Agrell, B.; Dehlin, O. The clock-drawing test. *Age Ageing* **1998**, *27*, 399–403, https://doi.org/10.1093/ageing/27.3.399.
48. Jenkinson, C.; Layte R. Development and testing of the UK SF-12 (short form health survey). *J. Health Serv. Res. Policy* **1997**, *2*, 14–18.
49. Montgomery, S.A.; Åsberg, M. A New Depression Scale Designed to be Sensitive to Change. *Br. J. Psychiatry* **1979**, *134*, 382–389, doi:10.1192/bjp.134.4.382.
